# Zipf’s law holds for phrases, not words

**DOI:** 10.1038/srep12209

**Published:** 2015-08-11

**Authors:** Jake Ryland Williams, Paul R. Lessard, Suma Desu, Eric M. Clark, James P. Bagrow, Christopher M. Danforth, Peter Sheridan Dodds

**Affiliations:** 1 Department of Mathematics & Statistics, Vermont Complex Systems Center, Computational Story Lab, & the Vermont Advanced Computing Core, The University of Vermont, Burlington, VT 05401; 2 Department of Mathematics, University of Colorado, Boulder CO, 80309; 3 Center for Computational Engineering, Massachusetts Institute of Technology, Cambridge, MA, 02139.

## Abstract

With Zipf’s law being originally and most famously observed for word frequency, it is surprisingly limited in its applicability to human language, holding over no more than three to four orders of magnitude before hitting a clear break in scaling. Here, building on the simple observation that phrases of one or more words comprise the most coherent units of meaning in language, we show empirically that Zipf’s law for phrases extends over as many as nine orders of rank magnitude. In doing so, we develop a principled and scalable statistical mechanical method of random text partitioning, which opens up a rich frontier of rigorous text analysis via a rank ordering of mixed length phrases.

Over the last century, the elements of many disparate systems have been found to approximately follow Zipf’s law—that element size is inversely proportional to element size rank^1^,^2^ —from city populations^2^,^3^,^4^, to firm sizes^5^, and family names^6^. Starting with Mandelbrot’s optimality argument^7^, and the dynamically growing, rich-get-richer model of Simon^3^, strident debates over theoretical mechanisms leading to Zipf’s law have continued until the present^8^,^9^,^10^,^11^. Persistent claims of uninteresting randomness underlying Zipf’s law^8^ have been successfully challenged^9^, and in non-linguistic systems, good evidence supports Simon’s model^3^,^12^,^13^ which has been found to be the basis of scale-free networks^14^,^15^.

For language, the vast majority of arguments have focused on the frequency of an individual word which we suggest here is the wrong fundamental unit of analysis. Words are an evident building block of language, and we are naturally drawn to simple counting as a primary means of analysis (the earliest examples are Biblical corcordances, dating to the 13th Century). And while we have defined morphemes as the most basic meaningful ‘atoms’ of language, the meaningful ‘molecules’ of language are clearly a mixture of individual words and phrases. The identification of meaningful phrases, or multi-word expressions, in natural language poses one of the largest obstacles to accurate machine translation^16^. In reading the phrases “New York City” or “Star Wars”, we effortlessly take them as irreducible constructions, different from the transparent sum of their parts. Indeed, it is only with some difficulty that we actively parse highly common phrases and consider their individuals words.

While partitioning a text into words is straightforward computationally, partitioning into meaningful phrases would appear to require a next level of sophistication involving online human analysis. But in order to contend with the increasingly very large sizes and rapid delivery rates of important text corpora—such as news and social media—we are obliged to find a simple, necessarily linguistically naive, yet effective method.

A natural possibility is to in some way capitalize on n-grams, which are a now common and fast approach for parsing a text. Large scale n-gram data sets have been made widely available for analysis, most notably through the Google Books project^17^. Unfortunately, all n-grams fail on a crucial front: in their counting they overlap, which obscures underlying word frequencies. Consequently, and crucially, we are unable to properly assign rankable frequency of usage weights to n-grams combined across all values of n.

Here, we introduce ‘random partitioning’, a method that is fast, intelligible, scalable, and appropriately preserves word frequencies: i.e., the sum of sensibly-weighted partitioned phrases is equal to the total number of words present. As we show, our method immediately yields the profound basic science result that phrases of mixed lengths, as opposed to just individual words, obey Zipf’s law, indicating the method can serve as a profitable approach to general text analysis. To explore a lower level of language, we also partition for sub-word units, or graphemes, by breaking words into letter sequences. In the remainder of the paper, we first describe random partitioning and then present results for a range of texts. We provide supporting evidence and code for our paper in the Supplementary Information and in the paper’s Online Appendices at http://compstorylab.org/share/papers/williams2015a/.

## Text partitioning

To begin our random partitioning process, we break a given text *T* into clauses, as demarcated by standard punctuation (other defensible schemes for obtaining clauses may also be used), and define the length norm, *ℓ*, of a given clause *t* (or phrase, *s* ∈ *S*) as its word count, written *ℓ*(*t*). We then define a partition, *P*, of a clause *t* to be a sequence of the boundaries surrounding its words:


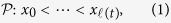


and note that *x*_0_, *x*_*ℓ*(*t*)_ ∈ *P* for any *P*, as we have (a priori) the demarcation knowledge of the clause. For example, consider the highly ambiguous text: “Hot dog doctor!”

Forgoing punctuation and casing, we might attempt to break the clause down, and interpret through the partition:


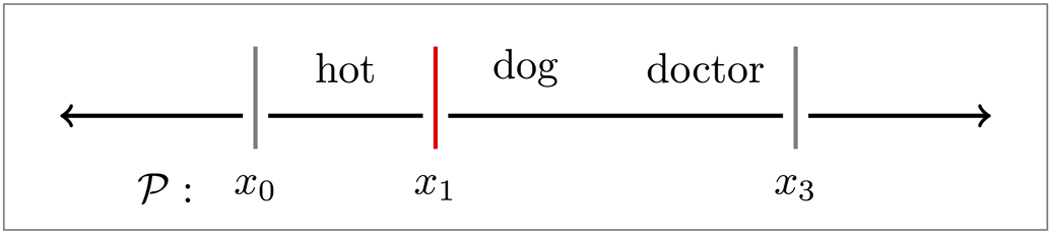


i.e., *P*  = {*x*_0_, *x*_1_, *x*_3_}, which breaks the text into phrases, “hot” and “dog doctor”, and assume it as reference to an attractive veterinarian (as was meant in Ref. 18). However, depending on our choice, we might have found an alternative meaning:


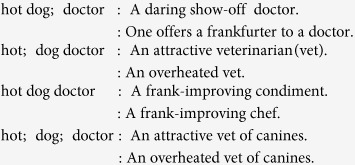


Note in the above that we (as well as the speaker in Ref. 18) have allowed the phrase “dog doctor” to carry synecdochic meaning in its non-restriction to canines, despite the usage of the word “dog”.

Now, in an ideal scenario we might have some knowledge of the likelihood for each boundary to be “cut” (which would produce an ‘informed’ partition method), but for now our goal is generality, and so we proceed, assuming a uniform boundary-cutting probability, *q*, across all *ℓ*(*t*) − 1 word-word (clause-internal) boundaries of a clause, *t*. In general, there are 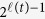 possible partitions of *t* involving 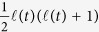 potential phrases. For each integral pair *i*, *j* with 1 ≤ *i *< *j* ≤ *ℓ*(*t*), we note that the probability for a randomly chosen partition of the clause *t* to include the (contiguous) phrase, *t*_*i…j*_, is determined by successful cutting at the ends of *t*_*i…j*_ and failures within (e.g., *x*_2_ must *not* be cut to produce “dog doctor”), accommodating for *t*_*i…j*_ reaching one or both ends of *t*, i.e.,


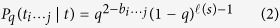


where *b*_*i…j*_ is the number of the clause’s boundaries shared by *t*_*i…j*_ and *t*. Allowing for a phrase *s* ∈ *S* to have labeling equivalence to multiple contiguous regions (i.e., *s* = *t*_*i…j*_ = *t*_*i*′…*j*′_, with *i, j* ≠ *i*′,*j*′) within a clause e.g., “ha ha” within “ha ha ha”, we interpret the ‘expected frequency’ of *s* given the text by the double sum:


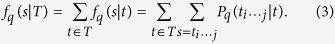


Departing from normal word counts, we may now have 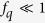, except when one partitions for word (*q* = 1) or clause (*q* = 0) frequencies. When weighted by phrase length, the partition frequencies of phrases from a clause sum to the total number of words originally present in the clause:


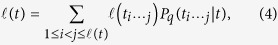


which ensures that when the expected frequencies of phrases, *s*, are summed (with the length norm) over the whole text:


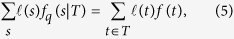


the underlying mass of words in the text is conserved (see SI-2 for proofs of Eqs (4) and (5)). Said differently, phrase partition frequencies (random or otherwise) conserve word frequencies through the length norm *ℓ*, and so have a physically meaningful relationship to the words on “the page.”

## Statistical Mechanical interpretation

Here, we focus on three natural kinds of partitions: *q* = 0 : clauses are partitioned only as clauses themselves; 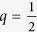 : what we call ‘pure random partitioning’ —all partitions of a clause are equally likely; and *q* = 1 : clauses are partitioned into words.

In carrying out pure random partitioning (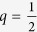), which we will show has the many desirable properties we seek, we are assuming all partitions are equally likely, reminiscent of equipartitioning used in statistical mechanics^19^. Extending the analogy, we can view *q* = 0 as a zero temperature limit, and *q* = 1 as an infinite temperature one. As an anchor for 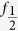, we note that words that appear once within a text—hapax legomena—will have 
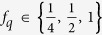
 (depending on clause boundaries), on the order of 1 as per standard word partitioning.

## Experiments and Results

Before we apply the random partition theory to produce our generalization of word count, *f*_*q*_, we will first examine the results of using the random partition process in a ‘one-off’ manner. We process through the clauses of a text once, cutting word-word boundaries (and in a parallel experiment for graphemes, cutting letter-letter boundaries within words) uniformly at random with probability 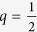.

**Figure 1:**
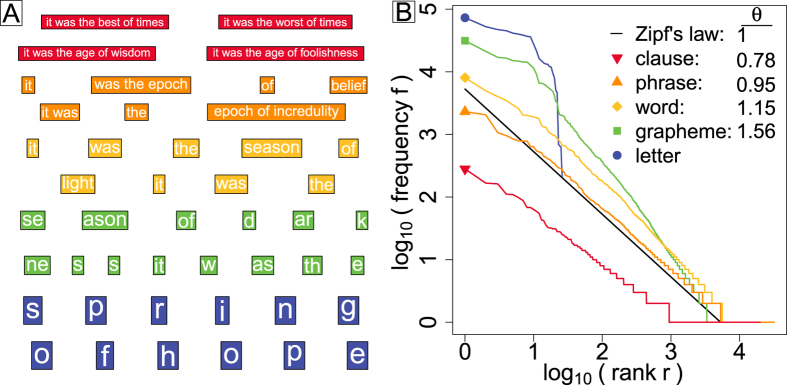
**A.** Partition examples for the start of Charles Dickens’s “Tale of Two Cities” at five distinct levels: clauses (red), pure random partitioning phrases (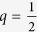, orange), words (yellow), pure random partitioning graphemes (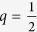, green), and letters (blue). The specific phrases and graphemes shown are for one realization of pure random partitioning. **B.** Zipf distributions for the five kinds of partitions along with estimates of the Zipf exponent *θ* when scaling is observed. No robust scaling is observed at the letter scale. The colors match those used in panel **A**, and the symbols at the start of each distribution are intended to strengthen the connection to the legend. See Ref. 28 and the Supplementary Information for measurement details.

In Fig. 1A we present an example ‘one-off’ partition of the first few lines of Charles Dickens’ “Tale of Two Cities”. We give example partitions at the scales of clauses (red), pure random partition phrases (orange), words (yellow), pure random partition graphemes (green), and letters (blue). In Fig. 1B, we show Zipf distributions for all five partitioning scales. We see that clauses (*q* = 0) and pure random partitioning phrases (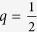) both adhere well to the pure form of *f* ∝ *r*^−*θ*^ where *r* is rank. For clauses we find 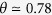 and for random partitioning, 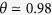 (see the Supplementary Information for measurement details and for examples of other works of literature). The quality of scaling degrades as we move down to words and graphemes with the appearance of scaling breaks^20^,^21^,^22^. Scaling vanishes entirely at the level of letters.

**Figure 2:**
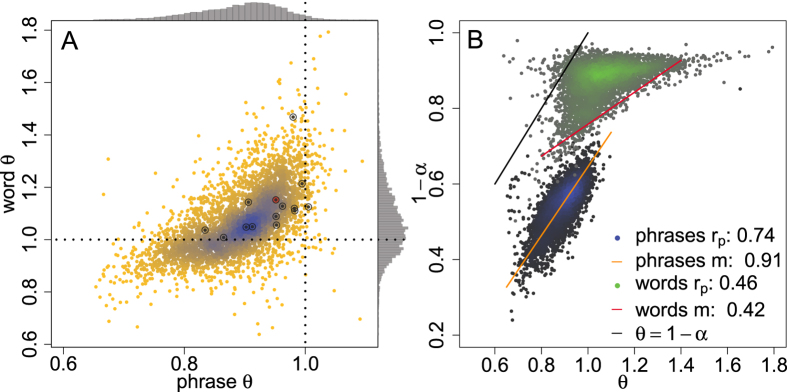
**A.**Density plot showing the Zipf exponent *θ* for ‘one-off’ randomly partitioned phrase and word Zipf distributions (q = 1 and q = 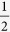) for around 4000 works of literature. We indicate “Tale of Two Cities” by the red circle, and with black circles, we represent measurements for 14 other works of literature analyzed further in the supplementary material. Marginal distributions are plotted as histograms along the edges of panel **A** and highlight how phrases typically exhibit θ  ≤ 1 whereas words produce unphysical θ > 1, according to Simons model. **B.** Test of the Simon model’s analytical connection θ = 1 − α, where θ is the Zipf exponent and α is the rate at which new terms (e.g., graphemes, words, phrases) are introduced throughout a text. We estimate α as the number of different words normalized by the total word volume. For both words and phrases, we compute linear fits using Reduced Major Axis (RMA) regression^24^ to obtain slope *m* along with the Pearson correlation coefficient r_p_. Words (green) do not exhibit a simple linear relationship whereas phrases do (blue), albeit clearly below the α = 1 − θ line in black.

Moving beyond a single work, we next summarize findings for a large collection of texts^23^ in Fig. 2A and compare the Zipf exponent *θ* for words and pure random 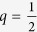 ‘one-off’ partitioning for around 4000 works of literature. We also plot the corresponding marginal distributions in Fig. 2A, and see that clearly 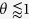 for 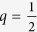 phrases, while for words, there is a strong positive skew with the majority of values of *θ* > 1. These steep scalings for words (and graphemes), *θ* > 1, are not dynamically accessible for Simon’s model^10^.

Leaving aside this non-physicality of Zipf distributions for words and concerns about breaks in scaling, we recall that Simon’s model^3^ connects the rate, *α*, at which new terms are introduced, to *θ* in a simple way: 1 − *α *= *θ*. Given frequency data from a pure Simon model, the word/phrase introduction rate is determined easily to be *α* = *N*/*M*, where *N* is the number of unique words/phrases, and *M* is the sum total of all word/phrase frequencies. We ask how well works of literature conform to this connection in Fig. 2B, and find that words (green dots) do not demonstrate any semblance of a linear relationship, whereas phrases (blue dots) exhibit a clear, if approximate, linear connection between 1 − *α* and *θ*.

Despite this linearity, we see that a pure Simon model fails to accurately predict the phrase distribution exponent *θ*. This is not surprising, as when *α* → 0, an immediate adherence to the rich-get-richer mechanism produces a transient behavior in which the first few (largest-count) word varieties exist out of proportion to the eventual scaling. Because a pure Zipf/Simon distribution preserves *θ* = 1 − *α*, we expect that a true, non-transient power-law consistently makes the underestimate 1 − *N*/*M* < *θ*.

**Figure 3:**
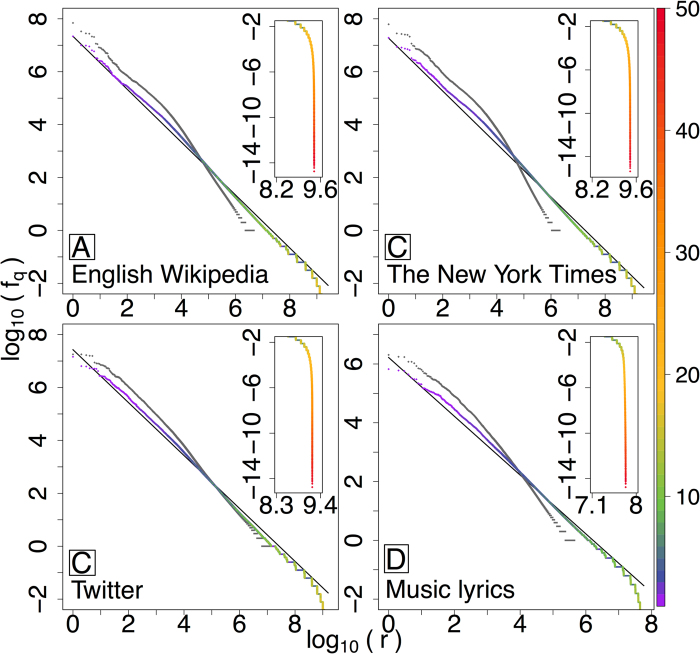
Random partitioning distributions (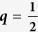) for the four large corpora: (A) Wikipedia (2010); (B) The New York Times (1987–2007); (C) Twitter (2009); and (D) Music Lyrics (1960–2007). Top right insets show the long tails of random partitioning distributions, and the colors represent phrase length as indicated by the color bar. The gray curves are standard Zipf distributions for words (*q* = 1), and exhibit limited scaling and with clear scaling breaks. See main text and Tabs. S1–S4, for example phrases.

Inspired by our results for one-off partitions of texts, we now consider ensembles of pure random partitioning for larger texts. In Fig. 3, we show Zipf distributions of expected partition frequency, *f*_*q*_, for 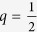 phrases for four large-scale corpora: English Wikipedia, the New York Times (NYT), Twitter, and music lyrics (ML), coloring the main curves according to the length of a phrase for each rank. For comparison, we also include word-level Zipf distributions (*q* = 1) for each text in gray, along with the canonical Zipf distribution (exponent *θ* = 1) for reference.

We observe scalings for the expected frequencies of phrases that hover around *θ* = 1 for over a remarkable 7–9 orders of magnitude. We note that while others have observed similar results by simply combining frequency distributions of n-grams^24^, these approaches were unprincipled as they over-counted words. For the randomly partitioned phrase distributions, the 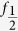, the scaling ranges we observe persist down to 10^−2^, beyond the happax legomena, which occur at frequencies greater than 10^−1^. Such robust scaling is in stark contrast to the very limited scaling of word frequencies (gray curves). For pure word partitioning, *q *= 1, we see two highly-distinct scaling regimes exhibited by each corpus, with shallow upper (Zipf) scalings at best extending over four orders of magnitude, and typically only three. (In a separate work, we investigate this double scaling finding evidence that text-mixing is the cause^22^.)

For all four corpora, random partitioning gives rise to a gradual interweaving of different length phrases when moving up through rank *r*. Single words remain the most frequent (purple), typically beginning to blend with two word phrases (blue) by rank *r* = 100. After the appearance of phrases of length around 10–20, depending on the corpus, we see the phrase rank distributions fall off sharply, due to long clauses that are highly unique in their construction (upper right insets).

In the Supplementary Information, we provide structured tables of example phrases extracted by pure random partitioning for all four corpora along with complete phrase data sets. As with standard *n*-grams, the texture of each corpus is quickly revealed by examining phrases of length 3, 4, and 5. For example, the second most common phrases of length 5 for the four corpora are routinized phrases: “the average household size was” (EW), “because of an editing error” (NYT), “i uploaded a youtube video” (TW), and “na na na na na” (ML). By design, random partitioning allows us to quantitatively compare and sort phrases of different lengths. For music lyrics, “la la la la la” has an expected frequency similar to “i don’t know why”, “just want to”, “we’ll have”, and “whatchu” while for the New York Times, “the new york stock exchange” is comparable to “believed to have” (see Table S2).

## Discussion

The phrases and their effective frequencies produced by our pure random partitioning method may serve as input to a range of higher order analyses. For example, information theoretic work may be readily carried out, context models may be built around phrase adjacency using insertion and deletion, and specific, sentence-level partitions may be realized from probabilistic partitions.

While we expect that other principled, more sophisticated approaches to partitioning texts into rankable mixed phrases should produce Zipf’s law spanning similar or more orders of magnitude in rank, we believe random partitioning—through its transparency, simplicity, and scalability—will prove to be a powerful method for exploring and understanding large-scale texts.

To conclude, our results reaffirm Zipf’s law for language, uncovering its applicability to a vast lexicon of phrases. Furthermore, we demonstrate that the general semantic units of statistical linguistic analysis can and must be phrases—not words—calling for a reevaluation and reinterpretation of past and present word-based studies in this new light.

## Methods

For the text analysis we perform here, we partition phrases from clauses, which we take to be sequences of words bounded by standard punctuation. We set all texts to lower-case and we consider words to be pure alphabetic sequences, allowing for two exceptions: apostrophes in between and at the end of alphabetic sequences, and hyphens strictly occurring within words.

We sourced works of literature from the Gutenberg Project^23^, using only those for which we could systematically remove preamble material. We obtained the English Wikipedia as its 2010 database dump^25^, the New York Times (1987–2007) from the Linguistic Data Consortium^26^, a random selection of 1/6th of the Twitter corpus from the standard “gardenhose feed” (typically 10% of all tweets), and music lyrics (1960–2007) as compiled for an earlier study of ours on emotion in written expression^27^. We provide all data at the paper's Online Appendices: http://compstorylab.org/share/papers/williams2015a/.

## Additional Information

**How to cite this article**: Williams, J. R. *et al.* Zipf’s law holds for phrases, not words. *Sci. Rep.* **5**, 12209; doi: 10.1038/srep12209 (2015).

## Supplementary Material

Supplementary Information
