# Supplementary material for: Zipf’s law holds for phrases, not words
Source: Sci Rep. 2015 Aug 11;5:12209. doi: 10.1038/srep12209 (PMC4531284; doi:10.1038/srep12209)
Supplement: Supplementary Information [file srep12209-s1.pdf]

Supplementary Information for **"Zipf's law holds for phrases, not words."**

Authors: Jake Ryland Williams, Paul R. Lessard, Suma Desu, Eric M. Clark, James P. Bagrow, Christopher M. Danforth, and Peter Sheridan Dodds.

SI-1: MATERIALS AND METHODS

To obtain the results in Fig. 2, we utilize the maximum likelihood estimation (MLE) procedure developed in [20]. In applying this procedure to clause and phrases distributions, several quantities are generally considered:

- $\hat{\theta}$: Zipf exponent estimate.
- $r_{\max}$: upper cutoff in rank r determined by MLE procedure.
- D: Kolmogorov-Smirnov (KS) statistic.
- p-value determined by the MLE procedure (note that higher is better in that the null hypothesis is more favored).
- $1-\alpha$: Estimate of Zipf exponent θ based on Simon's model [3] where α is the introduction rate of new terms. We estimate α as the number of unique terms (N) divided by the total number of terms (M).

which we report for 14 famous works of literature in SI-3.

In Fig. 2C we measure covariation between regressed values of $\hat{\theta}$ and the Simon model prediction $1-\alpha$. Since both are subject to measurement error ($\hat{\theta}$ is a regressed quantity and α is only coarsely approximated by N/M), we adhere to Reduced Major Axis regression [24], which produces equivalent results upon interchanging x and y variables, and hence guarantees that no information is assumed or lost when we place $\hat{\theta}$ as the x-variable).

To produce the rank-frequency distributions in Fig. ?? and words in tables S1–S4, we apply the random partition process to several large corpora from a wide scope of content. These corpora are: twenty years of New York Times articles (NYT, 1987–2007) [28], approximately 4% of a year's tweets (Twitter, 2009) [30], music lyrics from thousands of songs and authors (Lyrics, 1960–2007) [29], and a collection of complete Wikipedia articles (Wikipedia, 2010) [27]. In Fig. 2 we also use a subset of more than 4,000 books from the Project Gutenberg eBooks collection (eBooks, 2012) [?] of public-domain texts.

SI-2: PROOF OF f_q WORD CONSERVATION

In the body of this document we claim that the random partition frequencies of the phrases within a text T conserve the text's underlying mass of words, M_T. This claim relies on the fact that the partition frequencies of phrase-segments, $t_{i\cdots j}$, emerging from a single clause, t, preserve its word mass, $\ell(t)$. We represented this by the summation presented (Eq. 4) in the body of this document, which is equivalent to, $f_q(S \mid t)E_S[\ell(s) \mid t]$, i.e., the total number of words represented by the frequency of appearance of all phrases generated by the q-partition:

$$\begin{aligned} f(S \mid t)E_S[\ell(s) \mid t] &= \sum_{s\in S} \ell(s) f_q(s \mid t) \\ &= \sum_{s\in S}\sum_{s=t_{i\cdots j}} \ell(t_{i\cdots j})P_q(t_{i\cdots j} \mid t) \\ &= \sum_{1\leq i<j\leq \ell(t)} \ell(t_{i\cdots j})P_q(t_{i\cdots j} \,|\, t), \end{aligned} \tag{6}$$

which we now denote by $M(S \mid t)$ for brevity. For convenience, we now let $n = \ell(t)$ denote the clause's length and observe that for each phrase-length $k < n$ there are two single-boundary phrases having partition probability $q(1-q)^{k-1}$, and $n-k-1$ no-boundary phrases having partition probability $q^2(1-q)^{k-1}$. The contribution to the above sum by all k-length phrases is then given by

$$2kq(1-q)^{k-1} + (n-k-1)kq^2(1-q)^{k-1}. \tag{7}$$

Upon noting the frequency of the single phrase (equal to the clause t) whose length is n, $(1-q)^{n-1}$, we consider the sum over all $k \leq n$,

$$\begin{aligned} M(S \mid t) &= (1-q)^{n-1} \\ &+ [2q + nq^2] \sum_{k=1}^{n-1} k(1-q)^{k-1} \\ &- q^2 \sum_{k=1}^{n-1} k(k+1)(1-q)^{k-1}, \end{aligned} \tag{8}$$

which we will show equals n. We now define the quantity $x = 1 - q$ (the probability that a space remains intact), and in these terms find the sum to be:

$$\begin{aligned} M(S \mid t) &= nx^{n-1} \\ &+ \left[2(1-x) + n(1-x)^2\right] \sum_{k=1}^{n-1} kx^{k-1} \\ &- (1-x)^2 \sum_{k=1}^{n-1} k(k+1)x^{k-1}. \end{aligned} \tag{9}$$

This framing through x affords a nice representation in terms of the generating function

$$f(x) = \frac{1 - x^{n+1}}{1 - x}, \tag{10}$$

which allows us to express the summations through derivatives of $f(x)$:

$$\begin{aligned} &\sum_{k=1}^{n-1} kx^{k-1} = f'(x) - nx^{n-1}, \text{ and} \\ &\sum_{k=1}^{n-1} k(k+1)x^{k-1} = f''(x), \end{aligned} \tag{11}$$

to find

$$\begin{aligned} M(S \mid t) &= nx^{n-1} \\ &+ \left[2(1-x) + n(1-x)^2\right] \left(f'(x) - nx^{n-1}\right) \\ &- (1-x)^2 f''(x). \end{aligned} \tag{12}$$

Substitution of the second derivative term

$$f''(x)(1-x) = 2f'(x) - n(n+1)x^{n-1} \tag{13}$$

then produces the reduced form:

$$\begin{aligned} M(S \mid t) &= n[f'(x)(1-x)^2 \\ &- (nx^{n+1} - (n+1)x^n)], \end{aligned} \tag{14}$$

into which we substitute the first derivative term

$$f'(x)(1-x)^2 = 1 + nx^{n+1} - (n+1)x^n, \tag{15}$$

to render

$$\begin{aligned} M(S \mid t) &= n[1+nx^{n+1} - (n+1)x^n \\ &- (nx^{n+1} - (n+1)x^n)] = n, \end{aligned} \tag{16}$$

which proves Eq. 4. Putting this together into a sum over all clauses, we see proof of Eq. 5 naturally follows:

$$\begin{aligned} \sum_{s \in S} \ell(s) f_q(s \mid T) &= \sum_{t \in T} \sum_{s \in S} \ell(s) f_q(s \mid t) \\ &= \sum_{t \in T} M(S \mid t) = \sum_{t \in T} \ell(t). \end{aligned} \tag{17}$$

SI-3: PARAMETERS FOR WELL-KNOWN TEXTS

Below are tables showing fits of Zipf's exponent, $\hat{\theta}$, for 14 famous works of literature, along with details of the maximum likelihood estimation (MLE) procedure in [20]. The quantities used in these table are described in SI-1, Materials and Methods.

A Tale of Two Cities

level	$\hat{\theta}$	$r_{\max}$	D	p-value	$1-\alpha$
clause	0.783	3	0.0124	0.961	0.176
phrase	0.951	3	0.00742	0.772	0.603
word	1.15	4	0.0077	0.811	0.925
grapheme	1.56	4	0.0146	0.359	0.986

Moby Dick

level	$\hat{\theta}$	$r_{\max}$	D	p-value	$1-\alpha$
clause	0.296	1	0.0192	0	0.154
phrase	0.902	3	0.0132	0.0626	0.576
word	1.05	7	0.00986	0.61	0.912
grapheme	1.42	13	0.0109	0.953	0.986

Great Expectations

level	$\hat{\theta}$	$r_{\max}$	D	p-value	$1-\alpha$
clause	0.301	1	0.0199	0	0.186
phrase	0.995	5	0.0164	0.225	0.622
word	1.21	4	0.00943	0.526	0.938
grapheme	1.66	3	0.0147	0.181	0.988

Pride and Prejudice

level	$\hat{\theta}$	$r_{\max}$	D	p-value	$1-\alpha$
clause	1	3	0.0204	0.911	0.172
phrase	0.983	3	0.0148	0.149	0.617
word	1.11	18	0.0201	0.662	0.947
grapheme	1.43	24	0.0226	0.698	0.989

Adventures of Huckleberry Finn

level	$\hat{\theta}$	$r_{\max}$	D	p-value	$1-\alpha$
clause	0.881	4	0.0192	0.977	0.197
phrase	0.98	3	0.0119	0.385	0.625
word	1.47	1	0.0183	0.83	0.94
grapheme	1.66	6	0.0239	0.203	0.987

Alice's Adventures in Wonderland

level	$\hat{\theta}$	$r_{\max}$	D	p-value	$1-\alpha$
clause	0.707	2	0.0198	0.711	0.191
phrase	0.906	2	0.0108	0.687	0.555
word	1.14	6	0.0353	0.105	0.899
grapheme	1.19	49	0.0338	0.972	0.975

The Adventures of Tom Sawyer

level	$\hat{\theta}$	$r_{\max}$	D	p-value	$1-\alpha$
clause	0.321	1	0.0208	0	0.188
phrase	1.01	6	0.0173	0.826	0.555
word	1.12	3	0.0162	0.108	0.893
grapheme	1.51	4	0.0134	0.683	0.978

The Adventures of Sherlock Holmes

level	$\hat{\theta}$	$r_{\max}$	D	p-value	$1-\alpha$
clause	0.308	1	0.0231	0	0.191
phrase	0.952	4	0.0093	0.892	0.586
word	1.09	9	0.0144	0.733	0.921
grapheme	1.44	12	0.0191	0.663	0.983

Leaves of Grass

level	$\hat{\theta}$	$r_{\max}$	D	p-value	$1-\alpha$
clause	0.486	2	0.00768	0.783	0.0717
phrase	0.865	3	0.00971	0.463	0.543
word	1.01	6	0.0095	0.78	0.886
grapheme	1.39	7	0.0131	0.692	0.981

Ulysses

level	$\hat{\theta}$	$r_{\max}$	D	p-value	$1-\alpha$
clause	0.34	1	0.0192	0	0.193
phrase	0.912	4	0.0062	0.854	0.551
word	1.05	5	0.00773	0.515	0.887
grapheme	1.48	4	0.00874	0.61	0.983

Frankenstein; Or, The Modern Prometheus

level	$\hat{\theta}$	$r_{\max}$	D	p-value	$1-\alpha$
clause	0.257	1	0.0121	0	0.0741
phrase	0.834	2	0.0085	0.55	0.532
word	1.04	5	0.0215	0.057	0.906
grapheme	1.31	12	0.019	0.682	0.982

Wuthering Heights

level	$\hat{\theta}$	$r_{\max}$	D	p-value	$1-\alpha$
clause	0.927	3	0.0217	0.751	0.178
phrase	0.952	7	0.0104	0.978	0.581
word	1.06	10	0.0163	0.533	0.917
grapheme	1.54	5	0.0165	0.345	0.984

Sense and Sensibility

level	$\hat{\theta}$	$r_{\max}$	D	p-value	$1-\alpha$
clause	0.274	1	0.0176	0	0.142
phrase	0.982	3	0.00945	0.611	0.614
word	1.12	20	0.017	0.907	0.946
grapheme	1.41	28	0.0264	0.584	0.989

Oliver Twist

level	$\hat{\theta}$	$r_{\max}$	D	p-value	$1-\alpha$
clause	0.93	3	0.0152	0.808	0.242
phrase	0.962	3	0.00945	0.439	0.622
word	1.13	8	0.0118	0.695	0.931
grapheme	1.52	7	0.0153	0.521	0.987

SI-4: PHRASE FREQUENCY TABLES

The following tables contain selected phrases extracted by random partitioning for the four corpora examined in the main text. We provide complete phrase lists in csv format along with other material online at: http://www.uvm.edu/storylab/share/papers/williams2014a/.

rank	order=1	order=2	order=3	order=4	order=5
1	the (21763834.00)	of the (1332433.25)	one of the (42955.88)	in the united states (8425.91)	years of age or older (3363.23)
2	in (9935182.25)	in the (1095178.50)	as well as (41878.69)	at the age of (5873.75)	the average household size was (1669.62)
3	and (9708982.00)	to the (443282.25)	the united states (37460.25)	a member of the (5534.50)	were married couples living together (1662.12)
4	of (9015261.00)	and the (404687.00)	part of the (23948.69)	under the age of (5287.88)	from two or more races (1530.73)
5	a (6458405.25)	on the (335456.00)	at the time (17591.44)	the end of the (5013.12)	at the end of the (1512.25)
6	to (5890435.75)	at the (308288.50)	the age of (17212.81)	at the end of (4780.31)	the median income for a (1251.14)
7	was (3290575.00)	for the (282949.75)	the end of (16135.31)	as well as the (3805.84)	the result of the debate (1123.98)
8	is (3203926.00)	he was (276889.75)	according to the (16111.19)	at the same time (3609.44)	of it is land and (863.06)
9	he (2583977.75)	it is (246804.50)	may refer to (15914.88)	years of age or (3375.91)	the racial makeup of the (854.42)
10	on (2577531.25)	with the (233894.38)	member of the (15805.50)	of age or older (3364.88)	has a total area of (847.59)
11	as (2520721.50)	as a (230830.62)	the university of (15243.00)	the population density was (3354.00)	the per capita income for (841.80)
12	for (2409743.75)	it was (209433.25)	a number of (14994.00)	the median age was (3332.41)	and the average family size (838.66)
13	with (2107098.50)	from the (202985.38)	in the early (14390.50)	as of the census (3325.94)	and the median income for (832.59)
14	by (2010245.50)	the first (177129.12)	as a result (14356.69)	households out of which (3290.84)	the average family size was (831.62)
15	it (1960890.50)	as the (172026.62)	a member of (13692.75)	one of the most (2952.97)	had a median income of (831.28)
16	from (1688878.50)	was a (153285.75)	in the united (13589.25)	people per square mile (2875.00)	of all households were made (830.97)
17	that (1616682.00)	in a (152800.25)	he was a (13201.88)	at the university of (2866.38)	at an average density of (830.95)
18	s (1588172.00)	to be (142233.38)	of the population (13129.81)	was one of the (2728.66)	males had a median income (830.89)
19	at (1574302.50)	one of (128960.50)	in order to (12507.44)	for the first time (2684.28)	housing units at an average (829.80)
20	his (1461713.50)	during the (128190.62)	was born in (11809.50)	the result of the (2675.75)	made up of individuals and (829.12)
21	this (1187743.00)	of a (126613.62)	end of the (11779.88)	has a population of (2658.84)	had children under the age (828.27)
22	an (1121850.50)	with a (120564.38)	in the late (11641.56)	on the other hand (2654.81)	someone living alone who was (827.98)
23	are (965128.75)	and a (117848.38)	also known as (11477.12)	as part of the (2650.53)	income for a family was (825.89)
24	or (962634.50)	such as (116356.12)	in addition to (11229.75)	of those under age (2626.69)	had someone living alone who (825.33)
25	were (894722.00)	united states (107440.38)	it is a (11059.50)	during world war ii (2600.66)	householder with no husband present (823.45)
26	also (771224.25)	as well (105543.38)	world war ii (11018.56)	of the united states (2591.84)	had a female householder with (816.72)
27	be (736999.75)	th century (102688.62)	such as the (10948.00)	the median income for (2504.88)	population was spread out with (813.75)
28	has (711456.75)	was the (102566.25)	the result was (10670.12)	as a result of (2403.53)	this is a list of (784.14)
29	after (699095.75)	that the (98832.00)	most of the (10051.62)	he was born in (2381.19)	were below the poverty line (761.86)
30	however (689592.50)	and was (93389.38)	as part of (9636.19)	to the united states (2366.12)	the united states census bureau (743.83)
31	who (678548.00)	there were (88907.25)	he was the (9630.62)	in new york city (2292.53)	of the population were below (735.98)
32	they (674922.00)	after the (86291.12)	due to the (9612.06)	in the summer of (2204.88)	according to the united states (734.53)
33	one (657238.50)	new york (84445.25)	some of the (9501.06)	at the time of (2114.16)	was a member of the (711.28)
34	she (628094.25)	citation needed (83924.50)	at the end (9428.75)	the rest of the (2013.94)	result of the debate was (692.72)
35	had (625329.50)	he is (82930.25)	a population of (9329.19)	in the united kingdom (1997.19)	hispanic or latino of any (671.59)
36	first (581708.50)	there are (81538.25)	it is the (9142.31)	as well as a (1862.66)	at the time of the (643.62)
37	their (565228.50)	and in (79643.25)	there is a (9116.25)	of the population were (1848.16)	he was a member of (640.23)
38	there (563650.50)	part of (79108.00)	new york city (8808.00)	the result was delete (1845.00)	it has a population of (583.19)
39	when (554108.75)	for a (78926.88)	years of age (8519.56)	as one of the (1833.75)	national register of historic places (571.44)
40	new (542938.25)	to a (75288.88)	members of the (8365.31)	a total area of (1827.31)	it had a population of (562.61)
41	i (541554.00)	the united (73217.62)	of the year (8232.00)	was a member of (1718.38)	this list is intended to (554.30)
42	its (540743.50)	has been (72469.62)	the city of (8122.94)	below the poverty line (1715.28)	it does not imply that (553.88)
43	may (501345.50)	according to (71437.38)	this is a (8075.19)	the racial makeup of (1708.22)	interest in adding the link (553.47)
44	have (501019.00)	of his (71418.38)	was the first (7792.12)	the per capita income (1692.88)	below a full report on (552.31)
45	th (499317.50)	for example (67477.38)	was one of (7664.56)	the average household size (1691.25)	or that the involved accounts (550.88)
46	her (490852.25)	the new (66748.88)	in the world (7551.44)	married couples living together (1681.62)	accounts are spamming the link (550.88)
47	years (472193.25)	and is (65799.75)	in the first (7480.19)	a median income of (1677.53)	the external link gets used (549.97)
48	m (470841.75)	the same (65643.25)	in new york (7475.44)	and the average family (1677.31)	the village has a population (541.09)
49	all (448565.75)	the city (64700.38)	the united kingdom (7361.56)	average household size was (1670.56)	village has a population of (538.23)
50	other (443913.00)	this is (61094.62)	in the u (7321.44)	median income for a (1668.47)	of the debate was delete (470.31)
100	under (265543.75)	who was (39346.88)	at the same (5320.88)	in adding the link (1106.97)	does not imply that involved (275.44)
150	because (181436.25)	and he (30431.00)	in the town (4118.00)	for a household in (831.50)	household in the town was (172.14)
200	games (136824.00)	who had (26478.88)	the poverty line (3444.69)	at the start of (677.09)	like some other vfd subpages (128.88)
250	still (114752.25)	the american (22596.00)	the soviet union (3232.75)	is located on the (551.88)	the united states department of (103.78)
300	great (98223.75)	the uk (19042.25)	in the region (2778.00)	a municipality in the (491.28)	in the first round of (91.98)
350	court (84906.00)	in late (16349.38)	name of the (2500.62)	afds for this article (422.00)	an archive of the discussion (84.06)
400	further (75941.25)	and are (14785.12)	the government of (2289.19)	one of the oldest (383.66)	income for the village was (78.66)
450	does (68667.00)	size was (13455.25)	as early as (2140.56)	the origin of the (343.66)	township has a total area (71.88)
500	wrote (60868.25)	less than (12458.00)	according to a (1982.56)	in the development of (320.78)	can be seen in the (66.02)
600	hit (51657.00)	place in (10748.25)	also refer to (1758.62)	the new york city (280.81)	of the new york times (56.72)
700	ground (44973.75)	he took (9618.50)	of the show (1622.31)	he studied at the (252.50)	archive of an rfd nomination (49.69)
800	lower (39646.75)	the canadian (8583.50)	so that the (1498.31)	in the fourth quarter (226.56)	of the church of england (44.61)
900	fall (34990.25)	republic of (7820.88)	of the french (1378.50)	played college football at (210.66)	peerage of the united kingdom (41.45)
1000	ad (31756.75)	with other (7171.38)	united states navy (1246.62)	city has a total (197.91)	fis nordic world ski championships (39.11)
1500	garden (21653.75)	from which (5251.62)	throughout the world (916.56)	at the outbreak of (154.88)	it is found in europe (30.12)
2000	ben (16336.25)	the need (4203.12)	of the oldest (753.75)	also part of the (125.28)	as a result of their (24.70)
2500	band's (12613.25)	formed the (3456.62)	a small village (640.88)	it was performed in (107.12)	the first game of the (21.09)
3000	modified (10278.00)	of information (2951.50)	season in the (562.62)	was entered into the (93.94)	the second single from the (18.69)
3500	md (8490.25)	system is (2609.38)	the highest point (501.00)	as late as the (84.00)	he became chairman of the (16.84)
4000	mythology (7228.25)	water and (2340.88)	team for the (455.06)	he served with the (77.25)	the most important of these (15.38)
4500	joan (6291.25)	in singapore (2118.25)	to the french (416.31)	majority of the population (71.12)	in burgundy in north-central france (14.12)
5000	politically (5492.50)	cold war (1922.25)	has an area (386.50)	old at the time (65.78)	station went on the air (13.20)
6000	ignored (4359.25)	be given (1641.62)	national association of (334.69)	claimed to be the (57.66)	the population has grown at (11.66)
7000	lexington (3519.25)	and perhaps (1437.00)	for two weeks (297.25)	there may be a (51.59)	the university of new brunswick (10.50)
8000	blackburn (2945.50)	much larger (1281.50)	cities in the (268.31)	advanced to the final (46.97)	of the church of st (9.58)
9000	eighteenth (2495.75)	the ride (1156.88)	with the american (244.19)	used to determine the (42.91)	whom he had two children (8.86)
10000	validity (2140.00)	bit of (1053.75)	birth to a (224.81)	of the songs on (39.78)	the new york court of (8.23)
15000	topical (1181.00)	of alcohol (726.62)	the australian national (162.31)	continued to serve as (29.50)	over the next three seasons (6.17)
20000	timeslot (765.75)	history is (556.75)	queen elizabeth i (127.94)	he was also chairman (23.97)	unsuccessful candidate for election in (5.03)
25000	wheatley (545.50)	the peruvian (451.38)	and a friend (107.12)	and asks him to (20.25)	during the making of the (4.31)
30000	epithelial (411.50)	can change (376.50)	it difficult for (92.19)	from george washington university (17.69)	during the two world wars (3.78)
35000	awakes (324.25)	footage from (324.50)	the march of (81.12)	with this surname include (15.75)	was the eldest child of (3.38)
40000	ruck (262.75)	marion county (283.88)	over the area (72.56)	please give a reliable (14.25)	the immigration and nationality act (3.06)
45000	verbandsliga (218.50)	a sculpture (252.88)	his father worked (65.56)	so in order to (13.06)	in new york during the (2.83)
50000	imageshack (185.25)	break with (227.12)	fire at the (59.88)	departed new york on (12.06)	and his family lived in (2.61)
60000	partito (140.50)	injured his (188.62)	for economic development (51.19)	were only able to (10.50)	the permanent court of arbitration (2.30)
70000	akatsuki (110.50)	ships had (160.88)	on the sky (44.88)	as an actress in (9.34)	was first known as the (2.05)
80000	salley (89.75)	various characters (140.12)	stanford university and (39.94)	there is some overlap (8.41)	the northeast end of the (1.86)
90000	huila (74.75)	the sodium (123.88)	though in a (36.06)	during the following decade (7.69)	the australian national rugby league (1.70)
100000	leaven (63.75)	the condemned (110.88)	other two were (32.88)	closely linked with the (7.06)	already at the age of (1.58)

TABLE S1. Example phrases for English Wikipedia extracted by random partitioning.

rank	order=1	order=2	order=3	order=4	order=5
1	the (19034045.00)	of the (922676.50)	the united states (48226.25)	in the united states (7162.22)	at the end of the (599.03)
2	a (8722183.25)	in the (778571.88)	one of the (34160.31)	at the same time (5127.59)	because of an editing error (556.81)
3	and (8175499.25)	he said (506762.62)	in new york (32747.94)	for the first time (3893.78)	the new york stock exchange (514.61)
4	of (7463223.50)	to the (321805.25)	the new york (19706.31)	the new york times (3282.12)	for the first time in (481.62)
5	to (7094522.25)	and the (312622.62)	as well as (19019.81)	in new york city (3036.69)	he is survived by his (478.02)
6	in (6553996.25)	for the (275765.75)	new york city (17266.12)	at the end of (2664.31)	is survived by his wife (454.94)
7	that (3251408.00)	at the (266174.25)	a lot of (14997.94)	the end of the (2560.50)	an initial public offering of (400.08)
8	for (2849787.25)	new york (234356.50)	some of the (12923.62)	a spokesman for the (2556.88)	by the end of the (391.30)
9	he (2720690.75)	in a (228202.25)	part of the (12009.06)	at the university of (2224.84)	the end of the year (354.31)
10	is (2668672.00)	to be (182396.25)	of new york (11626.38)	one of the most (2167.66)	the securities and exchange commission (340.56)
11	it (2252598.00)	with the (180261.50)	president of the (10928.75)	of the united states (2105.25)	for the first time since (328.12)
12	but (2134976.50)	that the (179624.88)	the end of (10895.50)	a member of the (2028.19)	for students and the elderly (298.50)
13	on (2102270.50)	it is (171736.38)	there is a (10682.38)	the rest of the (1907.81)	beloved wife of the late (292.89)
14	with (2090580.50)	from the (165015.00)	director of the (10320.38)	at the age of (1877.81)	he said in an interview (287.44)
15	at (2042863.25)	of a (161459.62)	it was a (10318.81)	to the united states (1832.50)	the dow jones industrial average (276.14)
16	as (1808659.75)	she said (160297.25)	as a result (10075.00)	in lieu of flowers (1794.28)	the executive director of the (270.16)
17	i (1626505.00)	by the (159916.25)	according to the (10053.56)	executive director of the (1718.41)	tonight and tomorrow night at (253.62)
18	by (1573509.50)	it was (159603.00)	in the last (9828.88)	the united states and (1653.31)	in the last two years (243.44)
19	his (1418411.25)	as a (146938.88)	the white house (9593.25)	is one of the (1549.75)	in the new york times (240.67)
20	from (1397015.25)	he was (146862.00)	in the united (9578.31)	of the new york (1541.53)	in the last few years (235.52)
21	who (1317491.75)	is a (142374.75)	the university of (9083.88)	by the end of (1524.62)	in the united states and (229.91)
22	an (1253617.50)	with a (135244.50)	there is no (9027.81)	as well as the (1447.84)	in the middle of the (228.61)
23	are (1179629.75)	and a (126899.75)	it is a (8987.25)	the chairman of the (1339.56)	there are a lot of (222.73)
24	they (1177411.75)	but the (120749.75)	the first time (8735.56)	he is survived by (1330.34)	at the university of california (222.31)
25	not (1163949.50)	one of (118009.62)	in the first (8607.00)	the new york city (1322.84)	the federal bureau of investigation (221.33)
26	be (1140990.25)	for a (113570.88)	a spokesman for (8528.75)	in a telephone interview (1289.75)	the museum of modern art (220.48)
27	this (1017793.00)	the new (107764.88)	at the time (8300.88)	at a news conference (1162.12)	of the new york times (214.25)
28	which (985107.50)	the first (105144.75)	out of the (8246.56)	in the new york (1153.72)	graduated from the university of (210.23)
29	or (927178.00)	united states (103164.62)	in the past (8010.69)	for the most part (1147.06)	the food and drug administration (207.61)
30	new (892914.75)	as the (100548.38)	to be a (7877.38)	a son of mr (1144.06)	but at the same time (201.62)
31	had (865149.00)	is the (95388.62)	this is a (7856.44)	a spokeswoman for the (1103.06)	as a result of the (200.59)
32	one (826293.50)	will be (94356.50)	for the first (7789.44)	as a result of (1066.22)	the metropolitan museum of art (200.20)
33	about (820268.00)	to a (92111.75)	in an interview (7685.56)	a lot of people (1060.12)	the university of california at (193.88)
34	she (799892.00)	the united (91259.75)	he said he (7576.50)	a few years ago (1047.81)	years old and lived in (193.58)
35	s (796792.25)	there is (83281.62)	the number of (7551.12)	of new york city (1034.91)	for the new york times (189.27)
36	we (781654.50)	th street (81072.25)	of the new (7016.19)	new york stock exchange (1024.41)	received a master's degree in (179.23)
37	when (752716.25)	for example (74955.88)	the same time (6904.50)	at a time when (1023.19)	a memorial service will be (179.17)
38	will (704428.00)	according to (70748.12)	it was the (6859.56)	the director of the (1007.72)	new york and new jersey (176.58)
39	there (700976.25)	would be (70553.75)	it would be (6843.44)	survived by his wife (998.25)	president and chief executive of (175.53)
40	their (699595.50)	of his (70529.62)	in the world (6814.81)	as part of the (986.62)	president and chief operating officer (172.33)
41	p (687358.75)	this is (69945.38)	it is not (6789.88)	in the middle of (970.16)	at the time of the (167.44)
42	were (676437.25)	there are (69653.25)	in recent years (6653.56)	and the united states (956.59)	the rest of the world (165.12)
43	years (672249.00)	that he (69545.88)	in the early (6652.31)	from the university of (916.47)	th street and amsterdam avenue (164.03)
44	would (664100.25)	he is (69104.00)	in addition to (6584.25)	i don't want to (901.09)	the end of the day (156.91)
45	you (616708.00)	they are (68165.50)	the united nations (6541.31)	in addition to the (897.94)	the united states court of (155.62)
46	its (611930.00)	years ago (66357.25)	at the same (6344.44)	the first time in (897.12)	for more than a decade (151.02)
47	if (608648.75)	when the (65028.62)	but it is (6272.62)	in an effort to (888.00)	this film is rated r (149.75)
48	her (571742.75)	in his (62736.00)	at the end (6264.12)	as well as a (883.31)	spoke on condition of anonymity (148.44)
49	all (568749.50)	who is (62527.25)	i don't think (6247.25)	in the first half (883.22)	court of appeals for the (148.30)
50	been (552982.75)	and mr (61636.88)	i don't know (6171.06)	president and chief executive (882.94)	in the last five years (147.31)
100	here (259618.25)	to have (44901.62)	executive director of (4271.94)	in the middle east (614.88)	he graduated from the university (111.53)
150	st (168117.75)	trying to (32876.25)	and chief executive (3384.31)	tens of thousands of (498.16)	the virus that causes aids (92.28)
200	information (133141.25)	kind of (26368.75)	not going to (2943.56)	the heart of the (425.56)	secretary of state george p (79.97)
250	young (108081.25)	where he (21971.38)	a long time (2503.81)	the first half of (387.38)	came to the united states (69.20)
300	enough (93902.75)	he did (19303.00)	vice president for (2282.38)	for a total of (347.19)	salt and pepper to taste (63.61)
350	county (79788.75)	to pay (17182.75)	declined to comment (2116.62)	time on the market (315.03)	the new york city opera (58.09)
400	tax (72699.25)	the west (15687.75)	would like to (1993.69)	salt and freshly ground (288.91)	in state supreme court in (53.38)
450	became (65631.00)	to come (14304.75)	to more than (1884.75)	the vast majority of (272.25)	who is in charge of (49.69)
500	doing (59774.25)	the soviet (13439.88)	to build a (1787.25)	he said it was (257.59)	at the university of wisconsin (47.52)
600	quarter (51948.25)	a more (11832.00)	would be the (1569.44)	new york city police (231.50)	the good news is that (42.91)
700	someone (44616.75)	in november (10500.62)	a part of (1436.62)	state supreme court in (211.69)	he is also survived by (39.03)
800	weekend (39540.00)	get a (9667.62)	in a new (1314.62)	they don't want to (194.69)	in the next five years (36.31)
900	plays (35724.50)	given the (8871.62)	but for the (1227.50)	the last several years (184.69)	that he not be identified (33.95)
1000	ask (32280.00)	to show (8172.12)	they would be (1141.31)	those of us who (174.66)	upper east side of manhattan (31.92)
1500	reduce (21437.25)	in late (5853.50)	who heads the (872.69)	of the same name (135.16)	i don't know what to (24.73)
2000	seventh (15906.00)	and up (4597.88)	ought to be (721.38)	will continue to be (111.94)	the democratic congressional campaign committee (20.64)
2500	expansion (12556.75)	why the (3791.38)	of the biggest (621.31)	of the iraq war (97.66)	in the second half and (17.94)
3000	importance (10172.50)	and get (3287.00)	believed to have (545.69)	in front of his (86.75)	a good place to start (16.02)
3500	andy (8297.75)	idea that (2869.38)	he has made (492.75)	it is unclear how (78.78)	of the foreign relations committee (14.52)
4000	assessment (7023.75)	due to (2576.38)	of the report (453.06)	original moldings and detail (72.62)	there's no question about it (13.31)
4500	rye (6046.50)	which may (2336.75)	which he was (417.50)	the second and third (67.31)	the book review last year (12.30)
5000	officiated (5247.00)	ceremony at (2147.50)	affected by the (387.06)	to a multiyear contract (62.81)	the first day of school (11.50)
6000	distinctive (4090.00)	while others (1826.88)	economist at the (340.38)	to pay more than (55.56)	we are unable to acknowledge (10.25)
7000	racist (3296.75)	day for (1604.50)	the number to (305.81)	trinity college in hartford (50.03)	it is a question of (9.31)
8000	cracked (2726.75)	long term (1428.25)	and i hope (278.56)	the results have been (45.59)	filed in state supreme court (8.56)
9000	shrine (2306.25)	three and (1294.75)	throughout the state (256.19)	in the last seven (41.94)	the company went public in (7.92)
10000	handel's (1978.75)	new generation (1181.38)	of the home (236.62)	that the police had (39.03)	if there is such a (7.41)
15000	forgo (1063.50)	states supreme (818.12)	there are fewer (175.75)	its way through the (29.50)	of economics at the university (5.64)
20000	fujitsu (666.75)	come at (627.62)	a room with (140.88)	the history of american (24.12)	that donations be made to (4.62)
25000	refrained (456.50)	north fork (508.38)	to explain to (118.50)	and mayor david n (20.56)	that the soviet union would (3.97)
30000	tree' (335.25)	to disarm (427.50)	going for it (102.44)	the best they can (18.12)	a former republican senator from (3.52)
35000	afrikaans (256.00)	close and (367.12)	out of character (90.56)	end zone for a (16.22)	the east and the west (3.17)
40000	rushers (201.75)	by louis (321.62)	a maze of (81.19)	it also plans to (14.75)	and does not want to (2.89)
45000	andrews's (162.25)	after hitting (285.50)	sit in a (73.88)	the new law will (13.50)	he said the white house (2.67)
50000	hearne (133.00)	candidate is (256.88)	eastern european countries (67.81)	confirmed that he had (12.53)	it was the first victory (2.48)
60000	inxs (94.50)	accounting standards (213.50)	to use for (58.38)	of people in this (11.00)	and this was one of (2.19)
70000	airships (69.75)	compensation and (181.75)	doing enough to (51.31)	as if it could (9.81)	until the end of world (1.97)
80000	wei-sender (53.75)	dairy farmers (157.50)	he had missed (45.88)	new jersey attorney general (8.88)	game in the eighth inning (1.78)
90000	willan's (42.75)	table tennis (138.88)	and special events (41.50)	this is a town (8.12)	pleaded not guilty to all (1.64)
100000	prosecutable (35.00)	caught with (124.00)	you are ready (37.88)	i can't say enough (7.50)	the end of the new (1.53)

TABLE S2. Example phrases for the New York Times extracted by random partitioning.

rank	order=1	order=2	order=3	order=4	order=5
1	http (14482019.75)	in the (196458.75)	new blog post (34056.56)	i just took the (5910.19)	i favorited a youtube video (1839.47)
2	com (6428552.75)	i am (157031.25)	check it out (18386.69)	e meu resultado foi (5061.88)	i uploaded a youtube video (1453.28)
3	i (6227774.25)	i just (141596.00)	i love you (15578.25)	other people at http (3254.81)	just joined a video chat (1185.88)
4	ly (5320341.75)	of the (140377.62)	i just took (15341.56)	check this video out (3243.06)	fiddling with my blog post (917.62)
5	the (5180235.50)	on the (137894.88)	live on http (14544.62)	just joined a video (2371.72)	joined a video chat with (813.86)
6	bit (5140992.75)	i love (137768.62)	i want to (13955.88)	a day using http (2061.75)	i rated a youtube video (642.88)
7	a (5044536.50)	i have (136816.38)	i need to (12812.56)	on my way to (2006.66)	i just voted for http (582.91)
8	to (4183208.25)	going to (121491.12)	i have a (12131.88)	favorited a youtube video (1842.59)	this site just gave me (581.73)
9	o (2747181.50)	i think (120492.75)	quiz and got (11955.56)	i favorited a youtube (1839.59)	add a #twibbon to your (472.08)
10	rt (2735865.00)	to the (105588.38)	thanks for the (11796.62)	free online adult dating (1659.81)	the best way to get (454.42)
11	and (2671876.00)	to be (103771.38)	what about you (10897.31)	a video chat with (1628.81)	just changed my twitter background (444.41)
12	tinyurl (2630837.25)	i was (92118.25)	i think i (10602.31)	uploaded a youtube video (1461.91)	a video chat at http (375.84)
13	you (2594872.75)	if you (89098.88)	i have to (10443.56)	i uploaded a youtube (1453.50)	photos on facebook in the (356.89)
14	is (2589257.50)	at the (85136.38)	how are you (9339.94)	video chat at http (1435.12)	check it out at http (351.31)
15	in (2278977.00)	i know (81260.50)	looking forward to (9084.25)	what do you think (1435.09)	own video chat at http (341.72)
16	it (2209243.75)	have a (81252.50)	acabo de completar (9008.31)	i am going to (1398.34)	s channel on youtube http (304.75)
17	me (2049880.50)	to get (79410.75)	i love it (8357.44)	if you want to (1359.84)	and won in #mobsterworld http (293.28)
18	d (2017195.75)	this is (78757.50)	a youtube video (8342.50)	i wish i could (1356.75)	live stickam stream at http (289.97)
19	my (1967677.25)	and i (78420.62)	to go to (8035.69)	just got back from (1344.84)	on facebook in the album (289.03)
20	of (1925590.25)	but i (77363.75)	of the day (8032.19)	at the same time (1310.53)	added myself to the http (275.00)
21	on (1888067.25)	are you (76166.25)	what'll you get (7927.12)	thanks for the rt (1302.22)	just added myself to the (274.16)
22	bitly (1814009.75)	it is (73377.25)	my daily twittascope (7900.69)	channel on youtube http (1284.31)	alot of followers using http (251.34)
23	s (1644797.75)	i need (71952.50)	if you want (7526.38)	have a great day (1262.06)	has just done a job (235.92)
24	n (1528713.00)	it was (70856.62)	going to be (7514.31)	joined a video chat (1186.28)	of followers check out http (232.44)
25	lol (1514066.50)	is a (68456.88)	i don't know (7512.19)	is going to be (1167.62)	i love you so much (228.77)
26	p (1439044.75)	i want (67746.75)	i wish i (7496.06)	trying to figure out (1145.16)	if you want to get (225.47)
27	that (1325029.75)	i don't (67579.62)	is going to (7426.94)	thanks for the follow (1090.50)	hey i just got alot (202.48)
28	at (1297915.75)	i can (67569.12)	going to bed (7393.75)	to your avatar now (1077.56)	mb and the humidity is (202.31)
29	just (1260488.00)	to go (67275.00)	one of the (7351.12)	what are you doing (1069.03)	more followers go to http (201.09)
30	u (1232849.00)	just voted (66498.25)	a lot of (7075.44)	can't wait to see (1031.53)	make your own video chat (200.94)
31	de (1217523.25)	thank you (65912.62)	i feel like (7008.06)	com twitter directory under (1011.69)	you should check this site (199.98)
32	lt (1189756.75)	want to (64412.75)	i just got (6909.56)	check it out http (1004.12)	site out if you want (197.48)
33	this (1086481.00)	listening to (63914.62)	i need a (6752.81)	i have no idea (947.03)	where the wild things are (197.03)
34	e (1076997.00)	in a (63833.88)	in the morning (6713.38)	add a #twibbon to (944.44)	o luansantanaevc liga para voc (190.50)
35	so (1069598.50)	right now (63056.50)	on my way (6635.31)	with my blog post (933.28)	joined a video chat at (186.12)
36	www (1061481.75)	to do (61255.25)	let me know (6630.00)	i don't want to (929.91)	getting ready to go to (186.09)
37	no (1042255.25)	have to (59581.12)	just took the (6078.25)	fiddling with my blog (924.09)	keep up the good work (183.22)
38	gt (974932.50)	is the (58904.38)	meu resultado foi (6037.75)	i need to get (893.50)	gets you tons of followers (181.47)
39	t (973331.25)	on my (58570.00)	can't wait to (5670.00)	i want to go (865.00)	i just become a member (178.52)
40	with (964515.25)	you are (57962.88)	to be a (5662.25)	just got home from (861.56)	am i the only one (174.88)
41	but (962587.00)	do you (57464.62)	just woke up (5596.50)	thank you so much (855.88)	let me know if you (174.84)
42	im (961024.75)	at http (56957.25)	i just voted (5560.06)	the rest of the (851.44)	if you trying to get (173.91)
43	now (942129.00)	i got (56806.25)	what do you (5274.19)	going to be a (848.88)	on my way to the (173.20)
44	do (940366.75)	need to (56329.25)	just joined a (5162.19)	the best way to (831.09)	your own video chat at (170.86)
45	m (935591.50)	vote too (56158.62)	i am so (5077.88)	i wish i was (817.91)	at the end of the (163.81)
46	have (870991.75)	the best (55650.75)	e meu resultado (5062.62)	the end of the (789.47)	on my way to work (163.31)
47	be (868701.00)	try it (52316.88)	in the world (4978.25)	check out this site (787.66)	trying to figure out how (163.19)
48	twitpic (836558.50)	will be (51355.38)	happy new year (4964.62)	i can't wait to (784.03)	looking forward to your tweets (158.67)
49	up (816280.25)	i will (50980.00)	getting ready to (4894.19)	i am listening to (760.44)	this is going to be (154.58)
50	what (809227.00)	took the (50614.88)	getting ready for (4842.00)	to go to bed (757.81)	i want to go to (154.06)
100	know (434311.75)	on twitter (35430.38)	go to the (3525.19)	of followers using http (517.44)	sign up free and get (102.12)
150	come (265721.25)	good night (25500.38)	to see you (2833.94)	boa noite a todos (420.22)	don't know what to do (87.19)
200	watch (195026.50)	i mean (20696.62)	are you doing (2347.06)	more for gemini http (358.44)	the end of the world (65.86)
250	music (154807.00)	of it (18114.25)	at the end (2019.00)	a lot of people (310.47)	you know you want to (58.53)
300	soon (131072.75)	get the (15471.12)	out of my (1846.62)	all of a sudden (281.06)	us has given loan amount (52.56)
350	tell (113651.00)	i saw (13997.00)	to get the (1718.12)	i miss you too (259.38)	has been updated on nicedealz (48.59)
400	id (98939.25)	at a (12575.50)	to make it (1603.75)	a member of this (236.84)	now playing on smooth sounds (44.88)
450	gotta (85315.75)	i might (11760.38)	what happened to (1474.69)	how to make money (223.25)	i love him so much (42.11)
500	ne (76840.25)	able to (10855.50)	on the radio (1385.44)	calling it a night (209.53)	a day it work great (39.33)
600	care (63429.75)	my hair (9497.50)	to hear that (1256.88)	what do you want (191.09)	learn the trick discovered by (34.69)
700	once (52620.75)	as the (8335.25)	first day of (1133.31)	thanks for the #followfriday (177.00)	i just snapped a new (31.94)
800	final (45764.00)	me out (7537.88)	day at work (1047.62)	going to bed early (159.59)	only a matter of time (29.42)
900	search (40726.00)	what u (6958.75)	all is well (957.31)	that would be a (148.81)	need to go to the (27.66)
1000	jackson (37068.25)	next year (6506.38)	be on the (906.00)	know what that means (139.56)	you want more followers check (26.00)
1500	program (24182.75)	but then (4861.50)	when you are (689.25)	is supposed to be (106.97)	just woke up from my (20.38)
2000	jones (17577.75)	reminds me (3908.38)	not the only (576.38)	to see you in (88.22)	made me laugh out loud (16.84)
2500	pengen (13684.50)	playing with (3222.12)	not sure i (495.19)	i feel for you (76.06)	my friend made this great (14.50)
3000	host (11228.75)	a person (2784.50)	they are the (435.19)	long way to go (67.12)	get in the way of (12.94)
3500	ghost (9481.00)	when a (2445.00)	there was no (386.44)	hope it gets better (60.88)	so sorry for your loss (11.73)
4000	chi (8112.25)	this a (2158.62)	and if i (350.12)	this minute was presented (55.94)	home for rent in houston (10.75)
4500	attempt (7138.25)	no homo (1952.12)	to do what (322.62)	i sound like a (52.03)	want to take a nap (9.97)
5000	strength (6254.00)	wanna get (1783.00)	i can just (298.00)	u know what i (48.66)	or however you spell it (9.33)
6000	andr (4908.00)	off your (1514.00)	don't know where (259.31)	best of luck to (43.06)	im going to take a (8.25)
7000	jeremy (3970.00)	a world (1312.62)	of things to (231.00)	will there be a (38.56)	medical and nursing staff wanted (7.44)
8000	domestic (3314.75)	em out (1177.38)	just voted demi (208.12)	what not to wear (35.31)	get more great followers at (6.81)
9000	aje (2837.00)	so wrong (1060.62)	yes you do (190.75)	you have a link (32.59)	it just me or are (6.31)
10000	fase (2468.50)	now following (966.38)	will be available (175.81)	to be in bed (30.25)	longs to be romantically entangled (5.91)
15000	lagunya (1421.00)	head off (662.12)	know each other (127.38)	will do my best (22.84)	it was good seeing you (4.47)
20000	bts (946.00)	lovin the (502.75)	is in need (101.44)	we should meet up (18.56)	a little bit of both (3.70)
25000	grandino (690.00)	more photos (407.75)	in the light (84.62)	join our site for (15.72)	going to see where the (3.19)
30000	treysongz (536.50)	jus had (342.38)	in this week (73.31)	a break from work (13.81)	this is a great site (2.80)
35000	helluva (438.00)	some guys (295.38)	too hot for (64.69)	would like to share (12.34)	i am so sorry i (2.50)
40000	woots (368.00)	very scary (259.12)	don't get mad (57.94)	have to stay in (11.16)	how bad can it be (2.28)
45000	combi (315.00)	red dwarf (230.75)	hard is it (52.69)	see some of you (10.22)	we are working on the (2.09)
50000	rfd (274.25)	recession proof (208.00)	little help from (48.25)	giving it a try (9.44)	to your sign today can (1.95)
60000	wwwstickamjp (217.00)	video converter (173.25)	i woulda been (41.50)	want to say i (8.22)	a long time ago i (1.72)
70000	casbah (180.00)	wonderful evening (148.12)	holidays to all (36.31)	vu all over again (7.31)	don't have enough on my (1.55)
80000	coolman (151.00)	via de (129.62)	min to get (32.38)	i thought of u (6.59)	but i can't take it (1.41)
90000	caius (123.25)	a share (115.00)	that's just sad (29.31)	lady gaga or beyonce (6.03)	my body wants to be (1.28)
100000	aliena (102.50)	class right (103.25)	eating a bowl (26.75)	i can't imagine that (5.56)	never do much of anything (1.19)

TABLE S3. Example phrases for Twitter extracted by random partitioning.

rank	order=1	order=2	order=3	order=4	order=5
1	i (668838.75)	in the (28174.25)	i love you (2556.75)	la la la la (514.06)	la la la la la (184.89)
2	you (600813.50)	and i (25040.88)	i don't know (2094.00)	i don't want to (315.31)	na na na na na (93.98)
3	the (576318.50)	i know (17993.00)	i want to (1750.06)	na na na na (281.78)	on and on and on (48.28)
4	and (440698.25)	you know (16977.75)	la la la (1449.50)	in love with you (237.28)	i want you to know (47.70)
5	to (330196.75)	i don't (16237.12)	i want you (1229.00)	i want you to (227.75)	you know what i mean (45.64)
6	me (305085.75)	on the (14977.12)	you and me (1159.00)	i don't know what (201.38)	don't know what to do (45.22)
7	a (301126.50)	if you (13856.62)	i don't want (1105.88)	i don't know why (187.59)	oh oh oh oh oh (40.80)
8	it (219505.25)	to me (13048.50)	i know you (1086.00)	oh oh oh oh (181.59)	da da da da da (40.41)
9	my (205611.00)	to the (12940.75)	i need you (1065.12)	i want to be (172.69)	do do do do do (40.02)
10	in (203916.25)	to be (12614.00)	and i know (1051.62)	know what to do (144.06)	one more chance at love (35.66)
11	that (150464.50)	i can (12372.12)	i don't wanna (914.00)	what can i do (141.41)	i don't want to be (35.38)
12	of (149402.75)	and the (11679.88)	i got a (904.25)	yeah yeah yeah yeah (138.19)	in the middle of the (34.66)
13	on (143576.50)	but i (11512.50)	i know that (903.00)	you don't have to (137.38)	i don't give a fuck (33.81)
14	your (135024.00)	of the (11239.88)	you know i (902.69)	i close my eyes (130.31)	yeah yeah yeah yeah yeah (33.05)
15	but (132235.00)	i can't (10372.88)	i can see (872.62)	you want me to (129.19)	i don't know what to (32.39)
16	all (124985.50)	for you (10147.75)	and i don't (844.81)	you make me feel (128.31)	all i want is you (31.78)
17	so (121375.75)	when i (10046.38)	in your eyes (844.06)	i just want to (128.00)	you know i love you (26.88)
18	no (116877.00)	come on (9924.25)	i don't care (832.06)	da da da da (123.78)	the middle of the night (26.73)
19	we (113865.25)	you can (9686.00)	and if you (825.94)	if you want to (123.06)	the rest of my life (26.34)
20	is (113375.25)	i got (9577.88)	the way you (824.94)	come back to me (121.56)	no no no no no (26.11)
21	for (108828.50)	in my (9473.12)	all the time (817.62)	in the middle of (119.16)	at the end of the (25.30)
22	oh (107477.25)	all the (9467.25)	na na na (790.38)	and i don't know (118.72)	i wanna be with you (22.77)
23	be (107432.75)	i want (9396.50)	don't you know (766.62)	let me tell you (117.66)	all i wanna do is (22.44)
24	love (104438.50)	that i (9190.88)	this is the (766.25)	give it to me (111.97)	no matter what i do (22.41)
25	it's (99026.75)	i am (9141.88)	can't you see (761.19)	you are the one (111.94)	the way you love me (21.42)
26	now (95016.75)	and you (9048.75)	you love me (753.44)	do do do do (111.28)	no matter what you do (21.36)
27	don't (94956.00)	i was (9028.12)	oh oh oh (749.56)	i love you so (111.16)	what you do to me (20.83)
28	yeah (92807.00)	tell me (8783.50)	i wanna be (744.50)	all i want is (109.81)	when i close my eyes (20.31)
29	when (91600.75)	like a (8614.12)	you know that (714.38)	how does it feel (109.69)	and i don't know why (20.09)
30	with (90323.75)	the way (8512.38)	you want to (709.62)	know what i mean (109.12)	let me be the one (19.86)
31	what (90190.50)	to you (8289.50)	you don't know (707.62)	no no no no (104.03)	the end of the day (18.64)
32	this (90120.00)	when you (8157.62)	in my heart (693.69)	to be with you (100.81)	in the name of love (18.50)
33	know (89600.00)	if i (7941.50)	you and i (691.50)	i don't wanna be (97.50)	lemme see you drip sweat (18.00)
34	like (84259.00)	in a (7893.38)	you make me (675.19)	and on and on (96.47)	i like the way you (17.91)
35	just (83346.75)	my heart (7882.88)	if you want (663.81)	the end of the (94.66)	it's been a long time (17.89)
36	baby (83182.75)	for me (7880.50)	yeah yeah yeah (662.38)	i wish i could (93.09)	till the end of time (17.67)
37	do (81926.00)	this is (7754.62)	don't want to (654.62)	don't give a fuck (92.94)	i wish that i could (17.61)
38	up (81529.00)	for the (7570.88)	want to be (624.56)	can you feel it (91.88)	if you want me to (17.47)
39	if (74941.25)	let me (7539.25)	in my life (622.44)	the way i feel (91.00)	see it in your eyes (17.20)
40	chorus (72833.00)	with you (7482.62)	if i could (619.25)	i don't know how (90.47)	no matter what they say (16.78)
41	can (67057.50)	i need (7424.62)	you know what (615.06)	gon play with it (90.00)	and i don't know what (16.73)
42	down (66636.75)	with me (7386.00)	what you want (605.19)	you know that i (89.84)	let me hear you say (16.70)
43	get (63408.50)	you are (7208.25)	i used to (604.88)	at the end of (89.38)	i look into your eyes (16.70)
44	time (62579.50)	i wanna (7083.00)	on and on (595.94)	can you hear me (89.06)	i love the way you (16.64)
45	out (62562.50)	what you (6949.00)	i see you (592.88)	want you to know (88.38)	and i don't want to (16.45)
46	go (62101.75)	love you (6900.38)	in the sky (587.75)	out of my mind (86.62)	when i think of you (16.38)
47	quot (61793.50)	the world (6774.62)	in the air (584.06)	i need to know (86.56)	i look in your eyes (16.31)
48	got (60347.00)	do you (6733.50)	what to do (577.12)	all i wanna do (84.03)	the end of the world (16.16)
49	one (59306.50)	from the (6679.88)	all night long (558.19)	on the other side (83.88)	when the sun goes down (16.11)
50	see (58662.50)	want to (6649.88)	i know i (557.00)	do you love me (83.72)	still in love with you (16.02)
100	that's (28709.75)	in love (4324.38)	i just want (441.88)	that you love me (61.00)	you want me to do (11.83)
150	always (17981.50)	i won't (3225.00)	make me feel (369.31)	take a look at (51.09)	the end of the line (9.78)
200	en (13668.25)	without you (2692.50)	for you to (308.31)	you make me wanna (43.81)	that's the way it goes (8.77)
250	side (10606.00)	when i'm (2280.75)	who i am (277.81)	the rest of my (39.50)	the way that you do (7.88)
300	words (8896.00)	so long (2050.12)	on the wall (254.81)	open up your eyes (36.66)	i want to see you (7.23)
350	coming (7424.50)	have a (1815.25)	no one else (236.19)	get out of my (34.09)	makes the world go round (6.59)
400	ground (6669.25)	that's the (1645.12)	that's what i (218.94)	i don't want no (31.16)	tell me what you need (6.22)
450	death (5688.75)	then you (1506.12)	come back to (206.38)	don't mean a thing (29.25)	hey hey hey hey hey (5.81)
500	slow (5006.25)	i try (1382.25)	just want to (194.12)	goes on and on (27.84)	my my my my my (5.44)
600	cut (3808.00)	here i (1196.62)	i see your (172.44)	me like you do (25.44)	hey ladies drop it down (5.00)
700	grow (3091.25)	love with (1066.25)	in the game (158.62)	in front of you (23.47)	don't know if i can (4.61)
800	shut (2569.75)	my hands (969.25)	not the same (145.62)	you broke my heart (21.91)	it's been so long since (4.30)
900	doo (2167.75)	i tell (879.75)	yes i am (134.25)	me what you want (20.78)	you were the only one (4.06)
1000	seven (1898.75)	s a (802.88)	it was the (126.00)	all that i want (19.69)	just the way it is (3.88)
1500	food (1140.25)	am the (562.75)	a whole lot (95.38)	i wanna thank you (15.59)	mean a thing to me (3.20)
2000	fields (776.75)	caught up (434.12)	give me love (79.25)	got nothing to say (13.09)	a shoulder to cry on (2.78)
2500	vie (575.50)	saturday night (352.00)	yes you are (68.12)	know that you can (11.62)	was it good for you (2.48)
3000	compromise (451.00)	of things (295.38)	all about the (60.12)	is how we do (10.34)	right round like a record (2.27)
3500	couch (363.00)	the white (254.50)	think you can (53.75)	joy to the world (9.38)	your love would be untrue (2.08)
4000	pu (301.25)	they see (223.75)	i can fly (49.00)	if i don't get (8.69)	he was the only one (1.94)
4500	collect (254.75)	we'll have (197.62)	you said you'd (44.81)	give it all to (8.09)	you that we won't stop (1.81)
5000	product (219.25)	you drive (179.12)	want to hold (41.25)	wanna get with you (7.59)	cut me down to size (1.72)
6000	whatchu (169.50)	where you're (149.50)	take a breath (35.94)	your eyes on me (6.78)	round the ole oak tree (1.56)
7000	battered (135.25)	a plane (128.62)	right here in (32.00)	i wish i may (6.19)	move on down the line (1.44)
8000	verloren (111.25)	step out (111.88)	of all that (29.00)	we can make love (5.69)	bow wow wow yippie yo (1.33)
9000	nt (93.25)	fuck what (99.12)	be waiting for (26.44)	who the fuck are (5.25)	to warm a lonely night (1.25)
10000	honda (79.75)	you should've (88.38)	that what you (24.38)	like a loaded gun (4.88)	ain't that what you said (1.19)
15000	fuma (43.75)	little angel (57.50)	i wouldn't mind (17.44)	it's better this way (3.75)	on christmas day in the (0.94)
20000	cooper (28.50)	the undertow (42.00)	the wrong place (13.69)	since she left me (3.09)	and let it all go (0.80)
25000	fishy (20.25)	a major (32.88)	for one last (11.38)	and maybe you can (2.66)	no matter how far away (0.70)
30000	illtown (15.25)	alright baby (27.00)	you should try (9.75)	it take to make (2.34)	t want french fried potatoes (0.62)
35000	ndelo (12.00)	loud enough (22.62)	never give it (8.56)	i came to bring (2.12)	what you gave to me (0.58)
40000	rees (9.75)	view mirror (19.50)	to me a (7.62)	things i'm gonna do (1.94)	love is out the door (0.53)
45000	metaphoric (8.00)	your concern (17.12)	roll roll roll (6.88)	gotta say too much (1.75)	non ci sono solo io (0.50)
50000	memorizing (6.75)	the cancer (15.25)	on the eyes (6.25)	lay on the floor (1.62)	set the floor on fire (0.48)
60000	ajai (5.00)	an' then (12.38)	keep my eye (5.31)	give up on yourself (1.44)	right here next to you (0.44)
70000	aleiki (4.00)	cats be (10.38)	no more runnin' (4.62)	there's only one god (1.31)	gates of the seven seals (0.38)
80000	saatanan (3.25)	blijf ik (8.88)	we'll show them (4.12)	skies from now on (1.19)	we don't even have to (0.38)
90000	sauber (2.75)	yo tell (7.75)	time you say (3.69)	it comes to that (1.09)	ooh when you walk by (0.34)
100000	mosques (2.25)	believe anymore (6.88)	seemed so right (3.38)	but if i leave (1.00)	van de kille stemmen die (0.31)

TABLE S4. Example phrases for Music Lyrics extracted by random partitioning.
